# Trichostatin a Protects Dendritic Cells Against Oxygen-Glucose Deprivation via the SRSF3/PKM2/Glycolytic Pathway

**DOI:** 10.3389/fphar.2018.00612

**Published:** 2018-06-11

**Authors:** Hongyun Jiang, Siwei Zhang, Tongtong Song, Xin Guan, Ruojin Zhang, Xia Chen

**Affiliations:** Department of Pharmacology, College of Basic Medical Sciences, Jilin University, Changchun, China

**Keywords:** trichostatin A, dendritic cells, antigen-presenting cells, immunity, OGD, hypoxia

## Abstract

Dendritic cells (DCs) are important to the immune system and are frequently recruited to hypoxic regions, especially during acute myocardial infarction (AMI). Emerging data indicate that histone deacetylase (HDAC) inhibitors possess immunomodulatory functions. We previously showed in a rat model of AMI that the HDAC inhibitor TSA improved tissue repair, and this was accompanied by increased DC infiltration in the infarct region, suggesting an important role of TSA in modulating DC functions. To study the potential modulatory effect of TSA on DCs, we exploited an *in vitro* model of hypoxia and glucose deprivation. Culturing of DCs in the presence of 200 nM TSA improved DC survival under hypoxia and glucose deprivation. However, on a phenotypic level, TSA induced the expression of the DC co-stimulatory molecules CD80 and CD86, decreased FITC-dextran uptake, and facilitated DC migration. Moreover, TSA altered cytokine secretion by reducing the pro-inflammatory cytokines IL-1β, IL-10, IL-12, and TGF-β. Furthermore, TSA treatment enhanced HIF-1α-dependent glycolytic gene expression and increased pyruvate kinase M2 by upregulating SRSF3. These results suggest that by TSA alters important DC functions under hypoxia and glucose deprivation, and that TSA is critical for DC function by modulating SRSF3-PKM2-dependent glycolytic pathways.

## Introduction

Dendritic cells (DCs) are prominent antigen-presenting cells with immunoregulatory functions that depend on maturation and activation, as indicated by the expression of costimulatory molecules (e.g., CD80 and CD86; Albert et al., 2001; Schmidt and Mescher, 2002; Joffre et al., 2009). DCs are critical for initiating immune responses via the uptake, processing, and presenting of antigens. Moreover, DCs modulate innate and adaptive immunity by producing cytokines (e.g., IL-1β, IL-10, IL-12, and TGF-β; Guermonprez et al., 2002). DC development requires histone deacetylase (HDAC) activity (Chauvistré et al., 2014), and once stimulated, have reduced expression of maturation markers and cytokines (Frikeche et al., 2012a). Moreover, HDAC inhibition blocks inflammatory DC development triggered by GM-CSF (Sebastián et al., 2008; Singh et al., 2010). Emerging data suggested that HDAC inhibitors have immunomodulatory functions, but how HDAC blocks DCs function under hypoxic conditions is not clear.

Trichostatin A (TSA) is a broad-range HDAC inhibitor that inhibits class I and II HDACs. Preclinical studies show that TSA has potent anticancer activity and despite investigations in cancerous cells, its effects on immune function and DCs are not well known. DCs are frequently recruited to hypoxic regions, especially during acute myocardial infarction (AMI). Our unpublished data showed TSA could increase DCs at the infarct border region, and TSA-treated rats had improved tissue morphology after AMI. We previously showed in a rat model of AMI that TSA improved tissue repair accompanied by increased DC infiltration in the infarct region, suggesting an important role of TSA in modulating DC functions. Thus, we studied the potential modulatory effect of TSA on DC functions using an *in vitro* model of hypoxia and glucose deprivation.

## Materials and methods

### Cell line and cell culture

DC2.4, a murine bone marrow-derived immortalized dendritic cell lines were purchased from Shanghai Huiying Biological Technology co., LTD (China). Cells were grown in PRMI-1640 medium (Gibco Company, city and state are required) supplemented with 10% fetal bovine serum (Biological Industries, city and state are required), penicillin (100 U/ml) and streptomycin (100 μg/ml). Cells were passaged at 80% confluence.

For hypoxia, cells were grown in a three-gas incubator (SANYO) with 1% oxygen, 5% CO_2_, and 94% nitrogen. For glucose deprivation condition, PRMI-1640 medium without glucose (#11879020, Gibco) was used.

### Reagents

The HDAC inhibitor Trichostatin A (TSA) was purchased from APEXBIO Technology (#A8183,USA).

### MTT assay

Proliferating DC2.4 cells were seeded in 96-well plates at 20,000 cells/well and were grown overnight. Then cells were treated as indicated for 4 h. MTT assay was performed by adding 20 μl MTT (5 mg/ml, PBS) and 2 h later, supernatants were removed and 200 μl DMSO was added. Absorption was measured at 570 nm with a microplate reader (Tecan, Maennerdorf, Switzerland).

### Flow cytometry analysis of DC2.4 maturation

DC2.4 cells with indicated treatments were collected and fixed with 4% paraformaldehyde for 10 min at RT. Cells were the resuspended in PBS containing 0.5% BSA, followed by surface staining with FITC anti-mouse CD80 (1:20 in PBS, #104705, BioLegend, San Diego, CA) and anti-mouse CD86 (1:50 in PBS, #105007, BioLegend, San Diego, CA) antibodies at 4°C in the dark. Cells were then quantified using flow cytometry using a BD Accuri C6 Plus (BD, Mountain View, CA) and analyzed using FlowJo software (Tree Star, Inc., USA).

### FITC-dextran uptake assay

FITC-dextran was used as an endocytic substrate for dendritic cells.DC2.4 cells were treated as indicated. Then cells were collected and resuspended in PBS containing 1 mg/ml FITC-dextran at 37°C for 1 h. Negative control cells were incubated with FITC-dextran at 4°C. Signals from FITC channel were then detected using flow cytometry.

### Scratch wound assay

Confluent DC2.4 cells were scratched using a pipette tip and treated as indicated. After 16 h, cells were fixed with 4% formaldehyde and images were taken using an Olympus IX53 inverted microscope (Olympus, Japan). Evaluation of pictures was made using Image J software (NIH, city and state are required). Migration was quantified as the ratio of the area covered with cells and the area of the cell-free wound.

### Cytokine, ATP and lactate content in cell culture supernatant

DC2.4 cells were treated pre-treated with TSA for 4 h. Then cells were grown under indicated conditions and 24 h later, cell supernatant was collected, centrifuged at 3,000 rpm for 10 min. Then IL-1β, IL-10, IL-12, and TGF-β were measured using mouse ELISA kits (Bioss, Beijing, China). ATP and lactate was measured with kits from Camacho (Shanghai) Biological Technology Co. Ltd.

### DNA transfection

Plasmids coding mouse SRSF3 and PKM2 shRNA were purchased from Changchun Sai Xin Biological technology co, LTD. DNA transfection were performed using Lipofectamine® 2000 Reagent according to the manufacturer's protocol. Briefly, 1 μl DNA plus 3 μl transfection reagent were mixed in 500 μl medium to form a DNA complex. Then, 15 min later, the mixture was added to cells and 4 h later, supernatants were replaced with fresh media and the cells were grown for 24 h before experiments were performed.

### Total RNA isolation

Total RNA isolation was performed using Trizol® Reagent according to the manufacturer's protocol. Briefly, after treatment, cells were washed twice with ice cold PBS. Then, 1 ml Trizol reagent was added and 5 min later, samples were transferred into an RNA-free Eppendorf tube and mixed with 200 μl chloroform. After centrifugation (12,000 rpm, 15 min), supernatants were transferred to a new set of tubes and RNA was precipitated with isopropanol and resuspended in RNA-free water.

### Reverse-transcription

Total RNA (1,000 ng) was reverse transcribed into cDNA using TransScript First-Strand cDNA Synthesis SuperMix (Transgen, Beijing, China). Reactions were carried out at 42°C for 30 min and were terminated at 85°C for 5 min.

### Semi-quantitative real-time PCR

Semi-quantitative real-time PCR was performed on an ABI 7300 Real-Time PCR system using TransScript® II Green One-Step qRT-PCR SuperMix. The following primers were used.

**Table:** 

**Gene**	**Forward (5′-3′)**	**Reverse (5′-3′)**
PKM2	GTCTGGAGAAACAGCCAAGG	CGGAGTTCCTCGAATAGCTG
PKM1	GTCTGGAGAAACAGCCAAGG	TCTTCAAACAGCAGACGGTG
GAPDH	AGCTTGTCATCAACGGGAAG	TTTGATGTTAGTGGGGTCTCG
HIF-1α	ACCTTCATCGGAAACTCCAAAG	CTGTTAGGCTGGGAAAAGTTAGG
Ldha	GCTCCCCAGAACAAGATTACAG	TCGCCCTTGAGTTTGTCTTC
HK2	TGATCGCCTGCTTATTCACGG	AACCGCCTAGAAATCTCCAGA
Tpi1	TGAGCCGTTTCCACCGCCCTATTA	GCTCCAACCATGAGTTTCCAGCCC
SRSF3	TCGTCGTCCTCGAGATGATT	CTCCTTCTTGGGGATCTGC

*GAPDH was used as housekeeping gene*.

Fluorescence was analyzed using ABI 7300 system software. Calculation of relative mRNA quantity was done according to methods published by Pfaffl (2001).

### Immunoblotting

Immunoblotting was performed as described in the literature (Liebl et al., 2015). The following antibodies were used: PKM1 (15821-1-AP, Proteintech, Rosemont, IL, USA), PKM (C-11) (sc-365684, Invitrogen, USA), and SRSF3 (Sigma-Aldrich, USA).

### Statistical analysis

Experiments were performed at least 3 times in duplicates/triplicates. Data are presented as mean ± S.E.M. To compare the differences of more than three groups, one-way ANOVA followed by Tukey's *post hoc* tests were used, and also individual student's *t*-tests were conducted using GraphPad Prism (version 5.04, GraphPad Software, Inc.) and *p* < 0.05 were considered significant.

## Results

### TSA improves DC2.4 cell survival under glucose and oxygen deprivation

**Figure 1:**
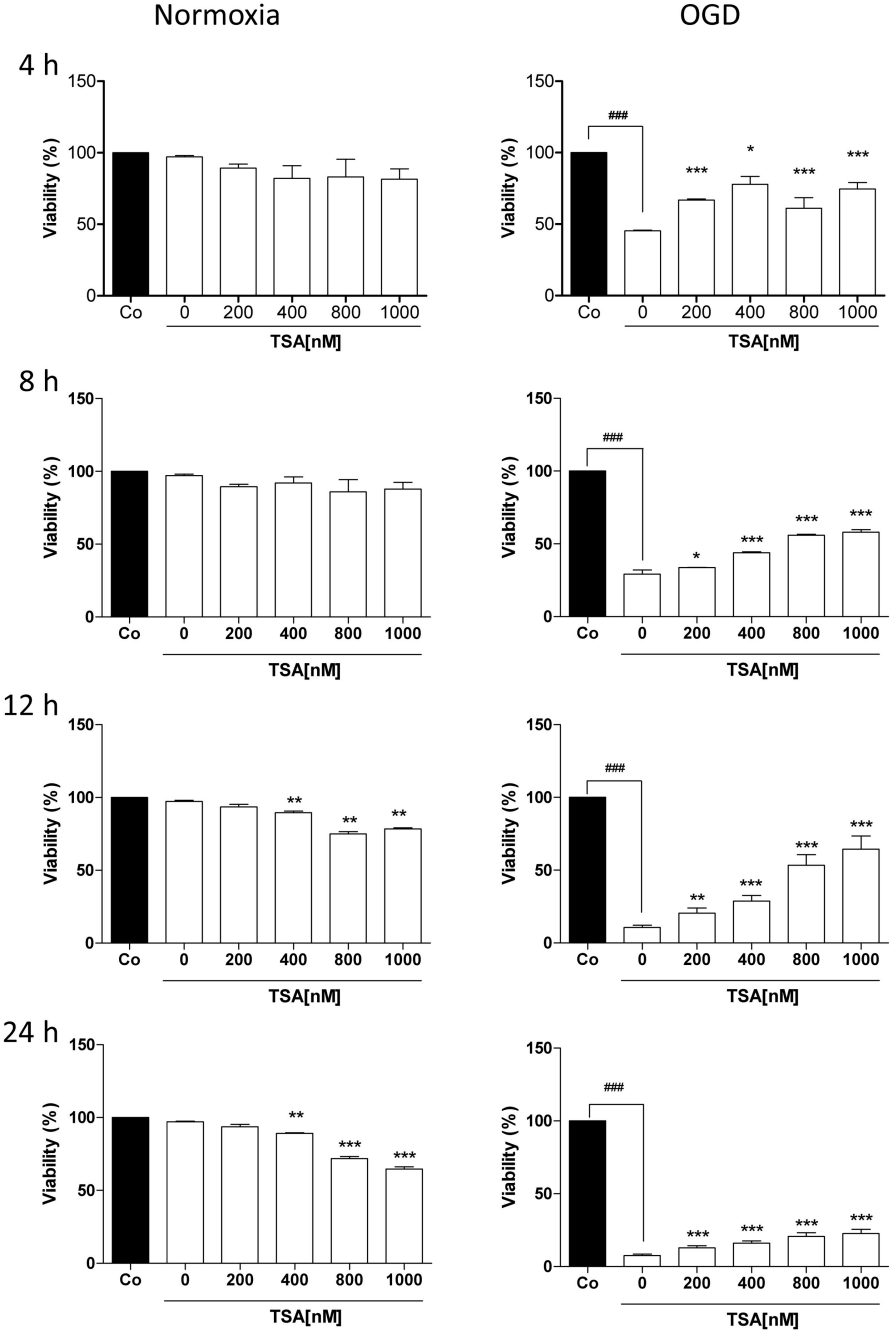
TSA improves DC2.4 cell survival under glucose and oxygen deprivation. DC2.4 cells were either left untreated or with treatment TSA for 4 h at indicated concentrations under normoxia (controls), glucose and oxygen deprivation (OGD). DC viability was assessed. ^*###*^*p* < 0.001, compared to controls. ^*^p < 0.05, ^**^p < 0.01, ^***^p < 0.001, compared to untreated group (TSA 0 nM), student's *T*-test.

We first performed MTT assay to determine suitable durations for glucose and oxygen deprivation and TSA dosages. As shown in Figure 1, TSA treatment (200 nM) had no apparent cytotoxic effects on DC2.4 cells from 4 to 24 h under normoxia. Under OGD condition, TSA treatment increased viability in a dose-dependent manner (*p* < 0.05, *p* < 0.001), showing protective effects. Therefore, we used TSA 200 nM and 4 h stimulation for the following experiments.

### TSA increases DC maturation

**Figure 2:**
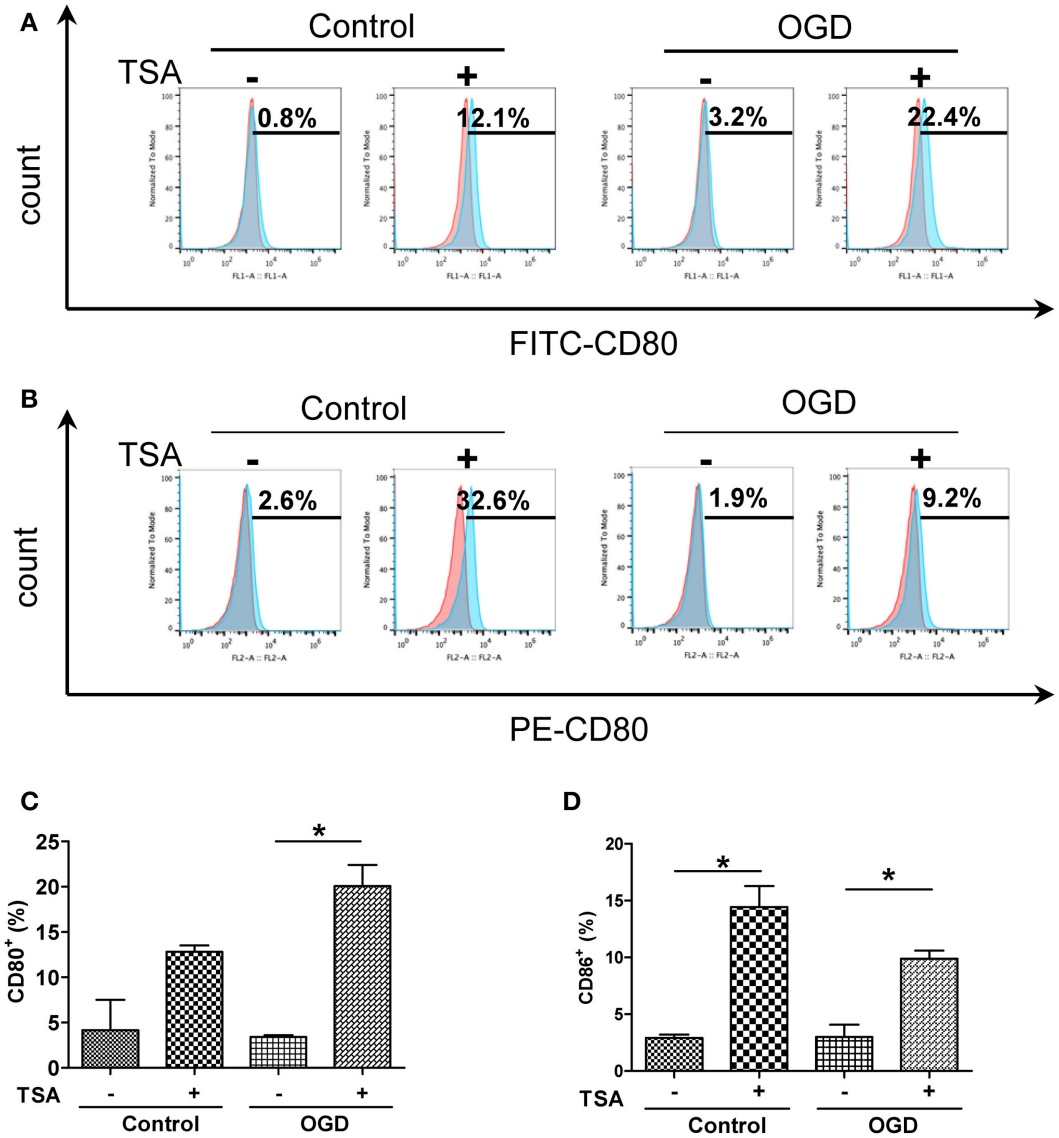
TSA increases expression of co-stimulatory molecules CD 80 and in DC2.4 cells under oxygen and deprivation. **(A,B)** Flow cytometry for surface expression of CD80 and CD86 in DC2.4 cells after TSA treatment under normoxia, glucose and OGD. Graphs represent three independent experiments. **(C,D)** Quantification of histograms from CD80^+^ and CD86^+^cells. ^*^p < 0.05.

**Figure 3:**
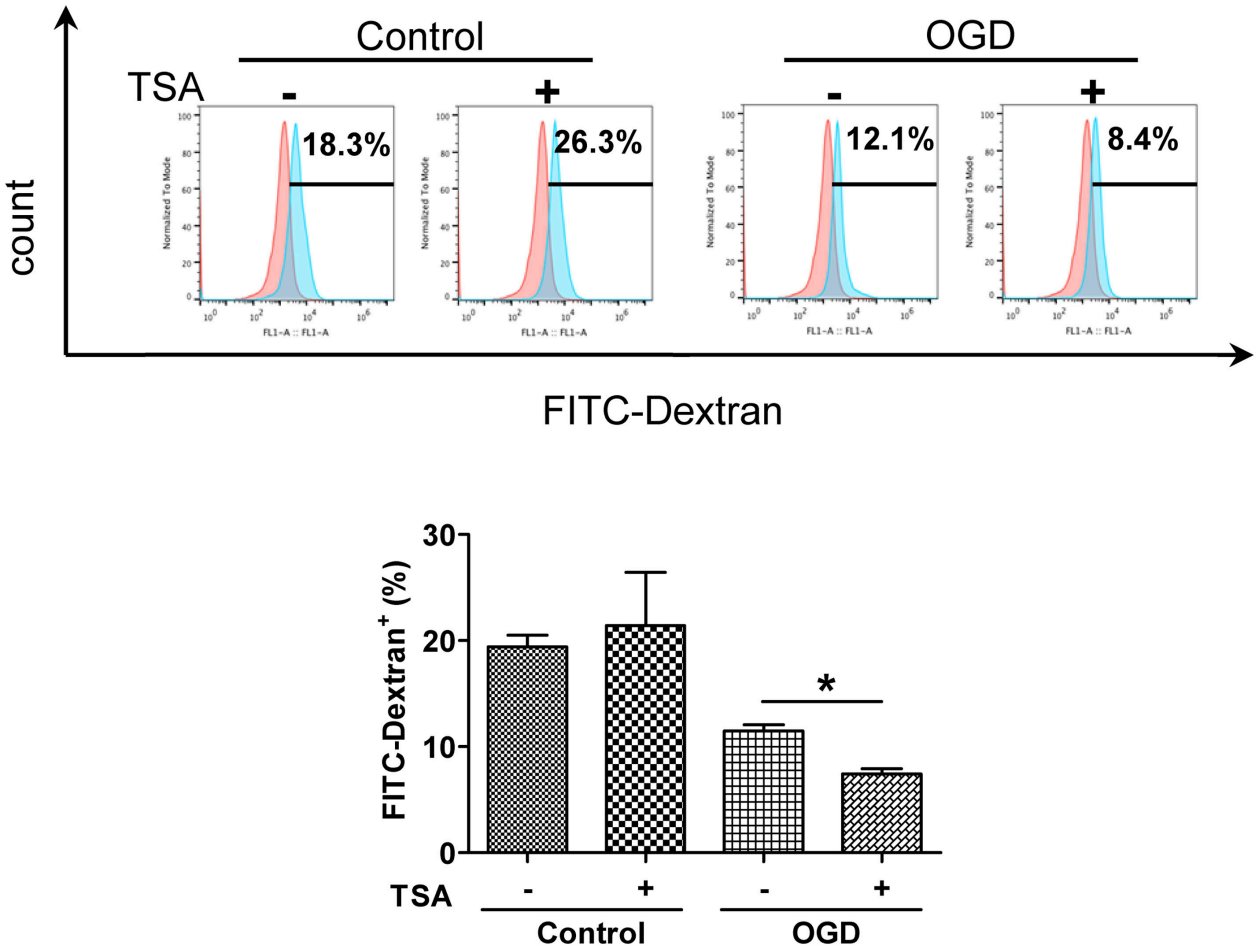
TSA decreases DC2.4 cell FITC-dextran uptake. Flow cytometry for FITC-dextran uptake by DC2.4 cells treated with TSA under normoxia, glucose and OGD. ^*^p < 0.05, compared to untreated controls.

Changes in oxygen and glucose had no apparent effect on basal expression of surface CD80 and CD86 expression (Figure 2). TSA treatment elevated CD86 expression under normoxia (*p* < 0.05) but CD80 was not affected. With OGD, TSA significantly increased CD80 and CD86 expression (*p* < 0.05). Also, FITC-dextran endocytic activity decreased with OGD (Figure 3). Thus, there is a maturation promoting effect of TSA with OGD.

### TSA improves DC2.4 cell motility

**Figure 4:**
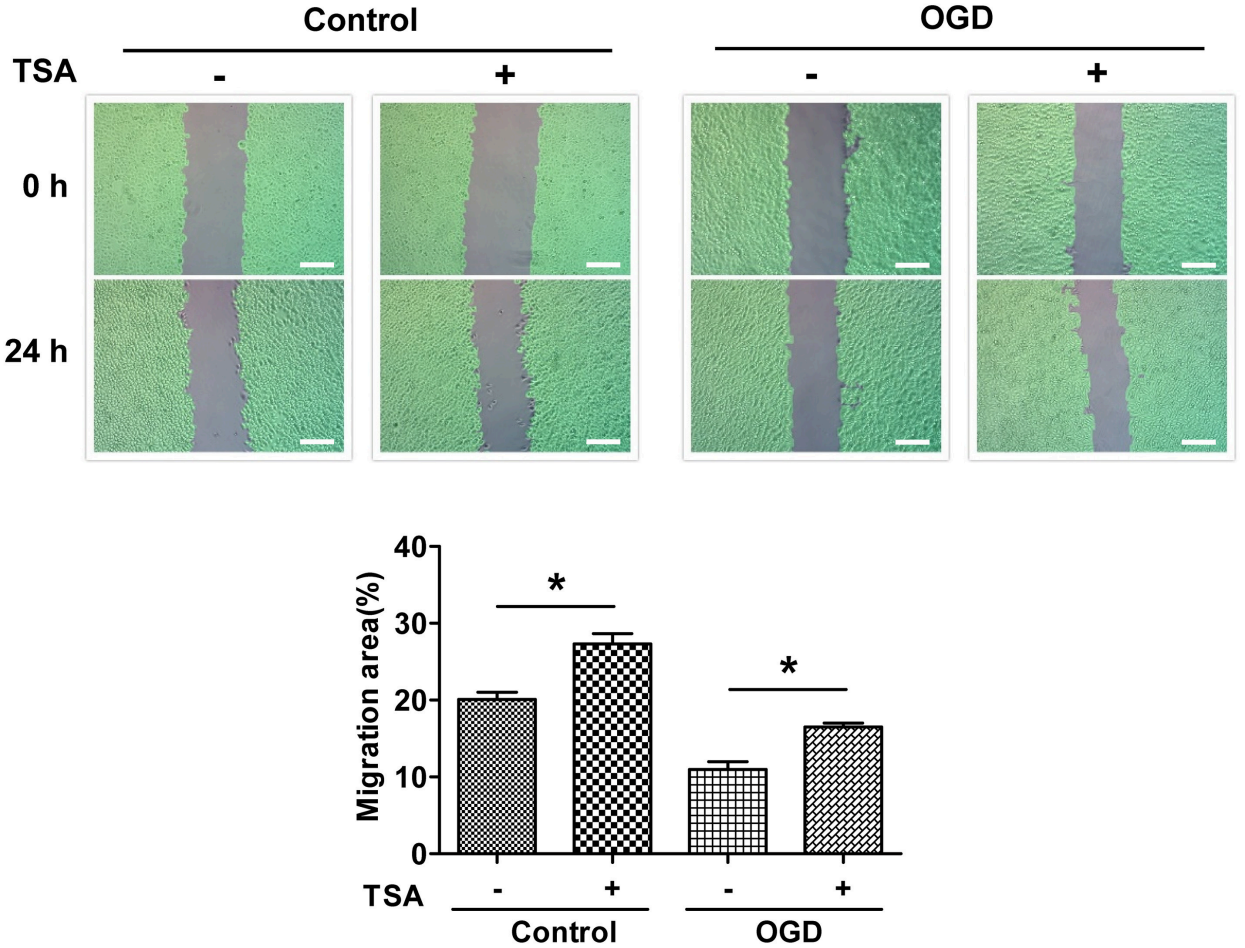
TSA promotes DC2.4 cell migration. Images of scratch wounds from untreated or TSA-treated DC2.4 cells (normoxia and OGD) after the scratch or after migration, ^*^p < 0.05, compared to controls. Green areas covered by cells.

As shown in Figure 4, TSA increased DC migration (*p* < 0.05) under normoxia and glucose-oxygen deprivation. These results indicate in general TSA could promote cellular motility.

### TSA decreases expression of proinflammatory cytokines in DC2.4 cells

**Figure 5:**
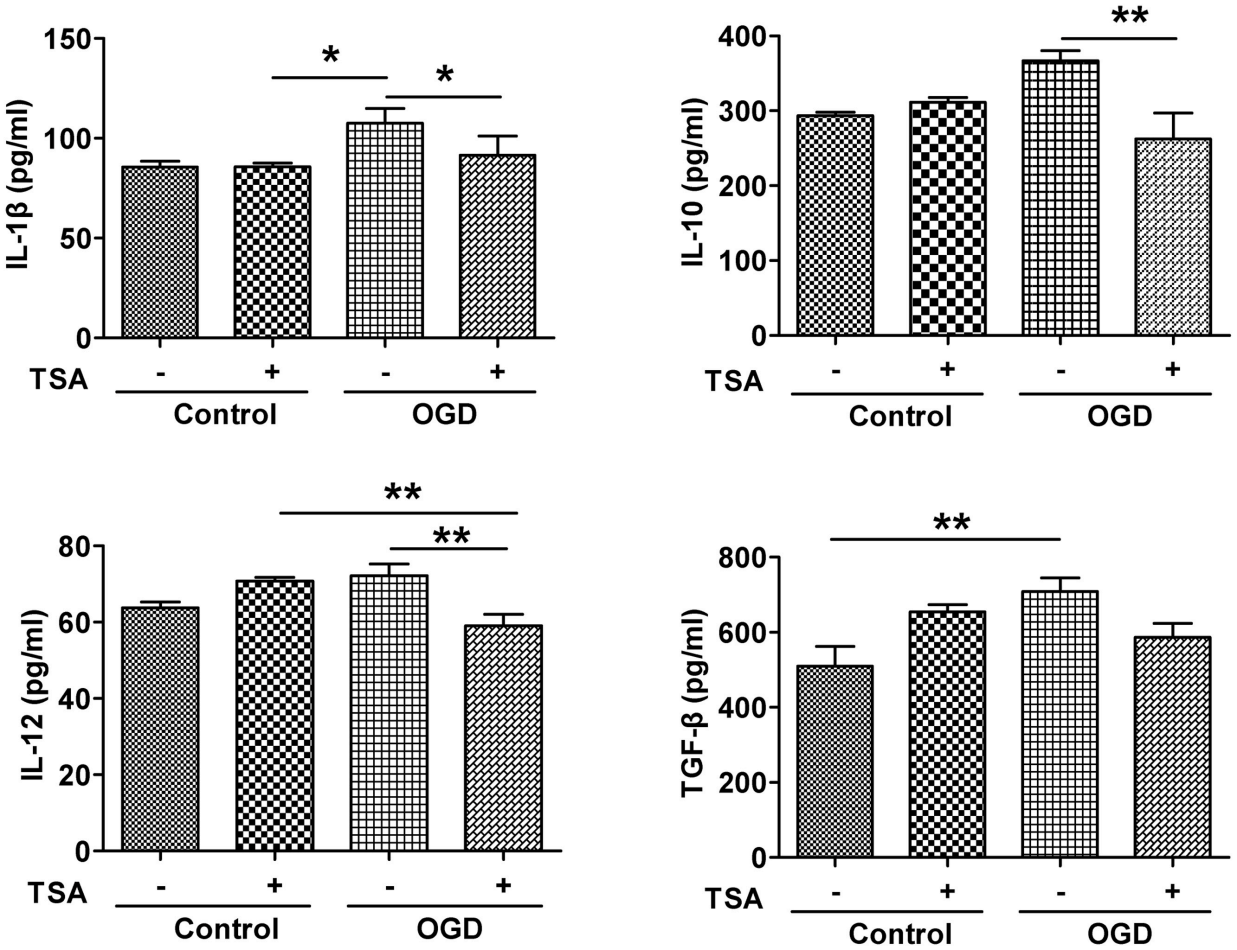
TSA decreases expression of IL-1β, IL-10, IL-12, and TGF- β in DC2.4 cells. Results are from three independent experiments. *N* = 3, ^*^p < 0.05, and ^**^p < 0.01.

Under normoxic conditions, TSA had no apparent effect on the expression of those cytokines (Figure 5). However, basal levels of these pro-inflammatory cytokines were elevated by OGD (Figure 5, upper panel). Surprisingly, TSA stimulation could reduce those cytokines (Figure 5). These results suggest TSA indeed could modulate cytokine under oxygen and glucose deprivation, possessing potential immunomodulating activity.

### TSA improves energy production by inducing expression glycolytic genes

**Figure 6:**
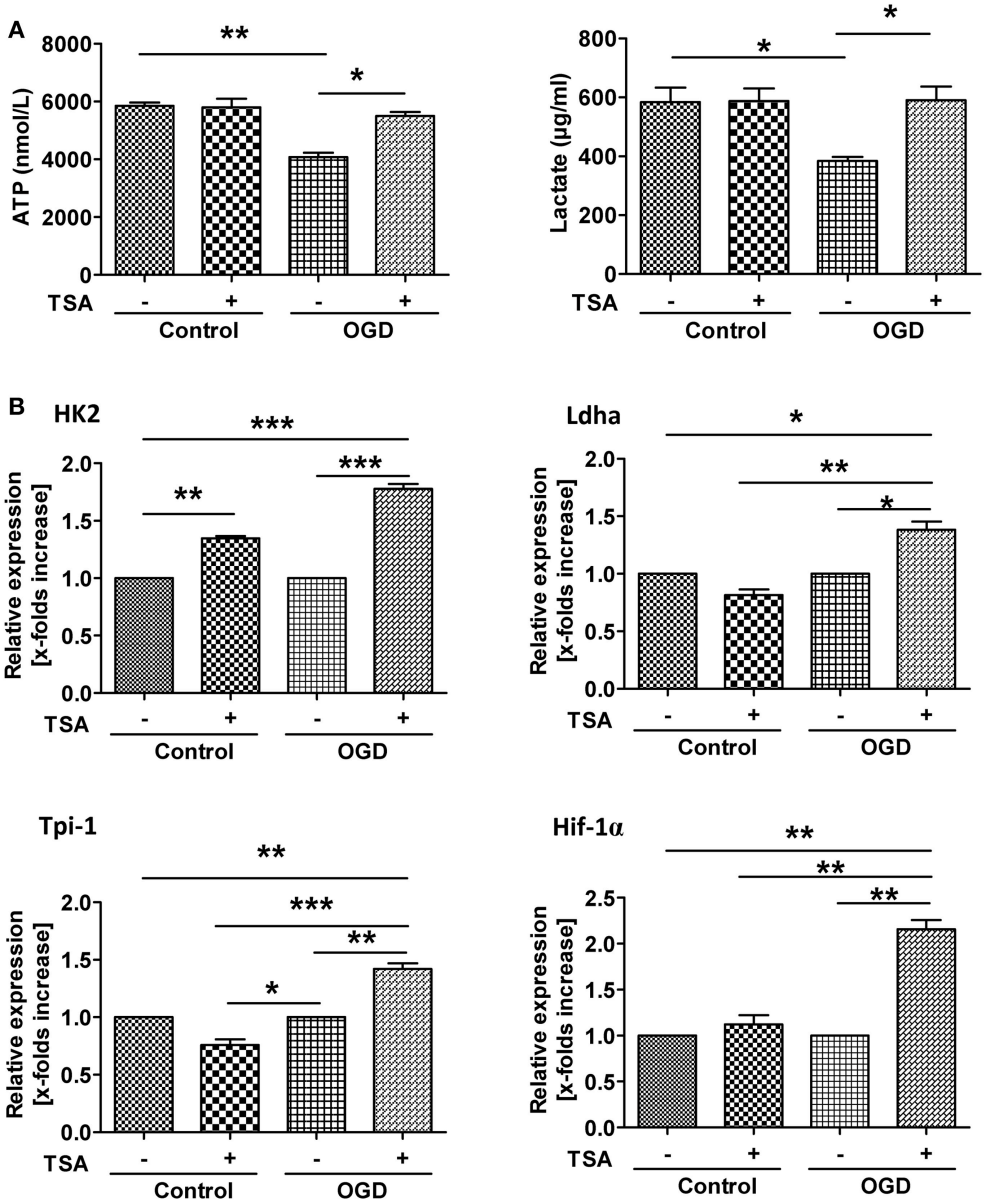
TSA improves energy production by inducing glycolytic gene expression. **(A)** ATP and lactate in cell culture supernatants. *n* = 3, ^*^p < 0.05, ^***^p < 0.01. **(B)** Glycolytic gene expression. *n* = 3, *p* < 0.05, ^**^p < 0.01, ^***^p < 0.001.

Under GD or OGD, ATP production were severely decreased (Figure 6A), indicating impaired energy production. Interestingly, TSA had no influence on ATP under normoxic conditions. However, under either oxygen or glucose deprivation, TSA treatment could increase ATP production. Under normoxia, TSA had no effect on lactate production. These results suggest TSA could benefit energy production under anaerobic conditions. These results, with the notion that TSA improves ATP production, clearly demonstrate the beneficial effects of TSA on glycolysis for ATP production. TSA significantly increased the expression of all four glycolytic gene under OGD, except for Ldha and Tpi-1 under normoxic condition (Figure 6B). These finding suggest that TSA benefit ATP production by upregulating glycolytic gene expression under oxygen and glucose deprivation.

### TSA promotes glycolysis by upregulating pyruvate kinase M2

**Figure 7:**
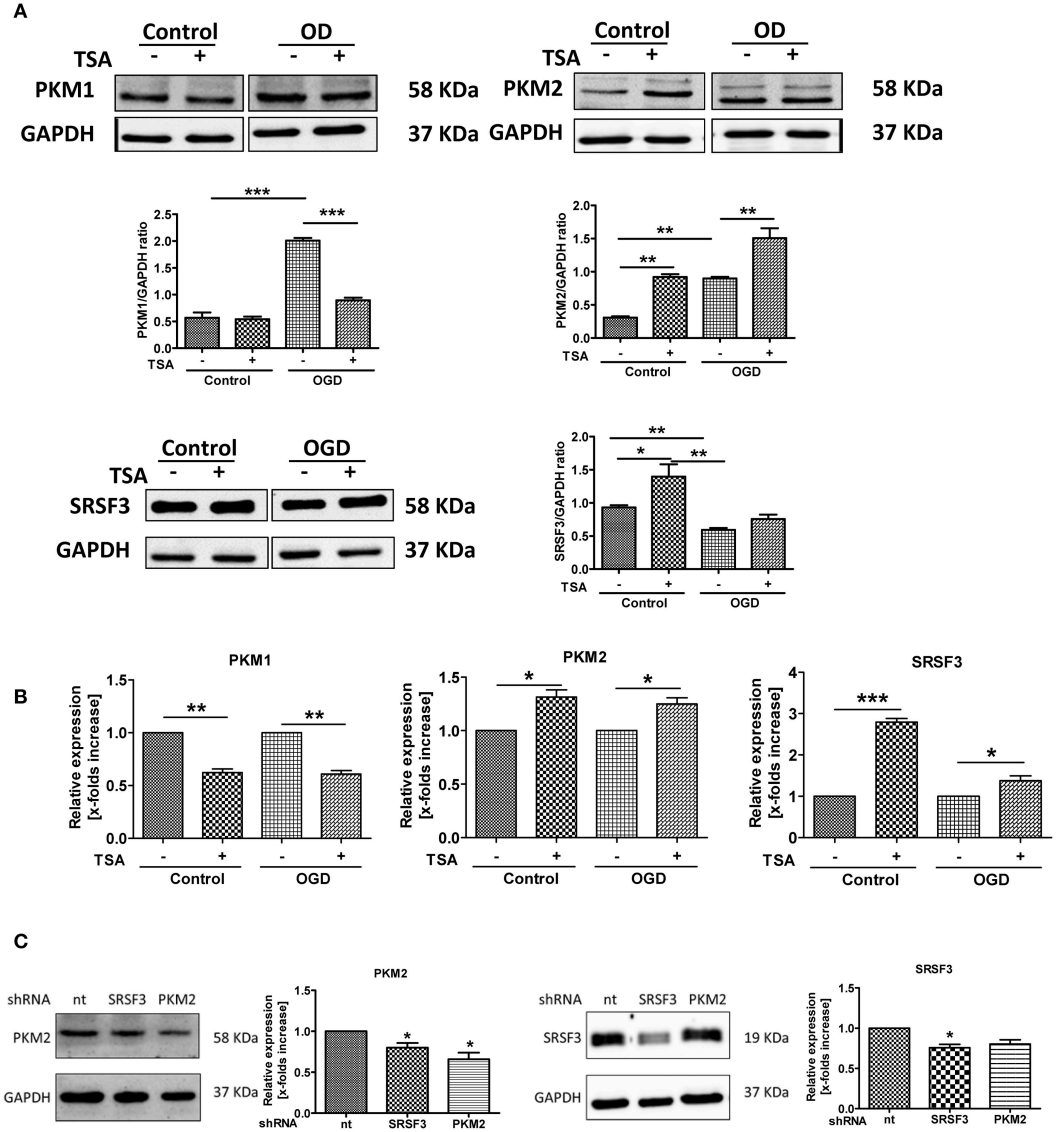
TSA promotes glycolysis by upregulating pyruvate kinase M2. **(A)** Protein expression and **(B)** mRNA for PKM1, PKM2 and SRSF3 from DC2.4 cells treated with TSA. *n* = 3, ^*^p < 0.05, ^**^p < 0.01, ^***^p < 0.001. **(C)** PKM2 and SRSF3 expression after silencing experiments. ^*^p < 0.05, compared to non-targeting control (nt).

As shown in Figure 7A, PKM1 and PKM2 expression were upregulated in response to OGD, indicating gene expression-mediated adaptations. However, TSA significantly downregulated PKM1 and increased PKM2 expression (Figure 7A). Thus, TSA may promote glycolysis by increasing PKM2 expression.

SRSF3 protein was slightly upregulated with OGD, and TSA increased SRSF3 protein and mRNA (Figures 7A,B). Silencing experiment data agreed. As shown in Figure 7C, silencing SRSF3 downregulated PKM2 expression and PKM2 knockdown had no influence on SRSF3 expression. Therefore, SRSF3 is clearly upstream of PKM2 and TSA may promote glycolysis through PKM2 by upregulating SRSF3 expression.

### Silencing of SRSF3 and PKM2 attenuates TSA induced glycolysis

**Figure 8:**
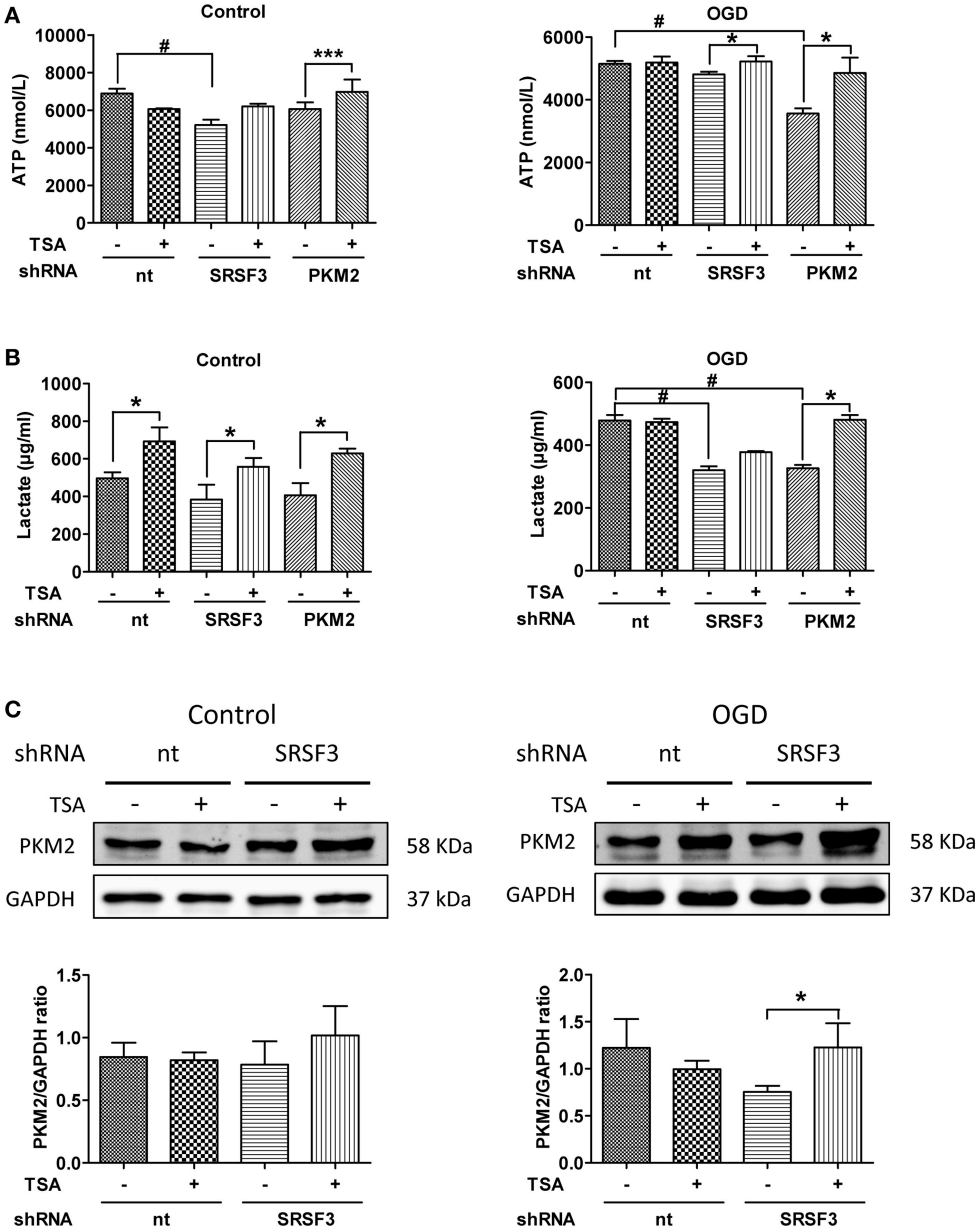
Silencing of SRSF3 and PKM2 attenuates TSA induced glycolysis. **(A)** ATP and **(B)** lactate from DC2.4 cells treated with TSA. ^*^p < 0.05, ^***^p < 0.001 compared with controls. ^#^*p* < 0.05 compared as indicated. **(C)** PKM2 expression from DC2.4 cells with SRSF3 silencing with/without TSA treatment. ^*^p < 0.05 compared to controls.

As shown in Figure 8A, under normoxic conditions, SRSF3 silencing reduced ATP production but PKM2 silencing had little effect. TSA increased ATP even when PKM2 was silenced. With OGD, silencing PKM2 reduced ATP production and silencing SRSF3 had no apparent effect. TSA increased ATP production when SRSF3 or PKM2 were silenced, suggesting that the SRSF3-PKM2 axis is involved in energy production effect of TSA.

With normoxia, lactate production was induced even when SRSF2 or PKM2 were silenced (Figures 8B,C), indicating the existence of an alternative compensating pathway. With OGD, SFSF3 silencing decreased lactate production, but TSA had little recovering effect (Figures 8B,C). The effect of TSA is only obvious when PKM2 is silenced, so SRSF3 is upstream of PKM2 and TSA favors glycolytic energy production via upregulation of SRSF3-PKM2 signaling.

### SRSF3/PKM2 pathway influences cytokine production in DCs

**Figure 9:**
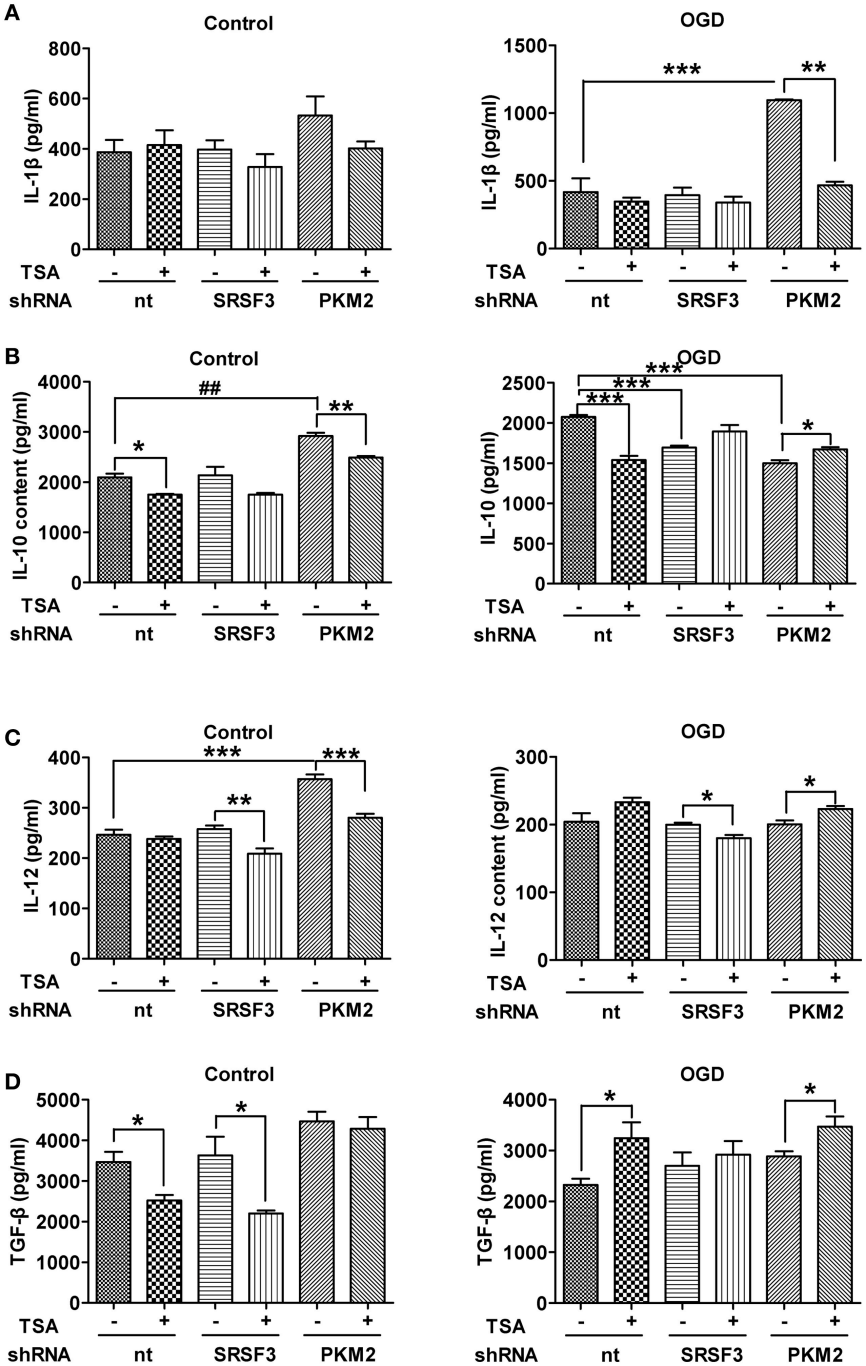
SRSF3/PKM2 pathway influences cytokine production in DCs. Cytokines in culture supernatant from DC2.4 cells with SRSF3 or PKM2 silencing, with/without TSA treatment. **(A)** IL-1β, **(B)** IL-10, **(C)** IL-12 and **(D)** TGF-β levels under indicated conditions are shown. ^*^p < 0.05, ^**^p < 0.01, ^***^p < 0.001, compared to controls.

As shown in Figure 9, with normoxia, silencing PKM2 induced expression of IL-10, IL12, but while IL-1β and TGF β partially increased. With OGD, PKM2 silencing induced expression of IL-1 β but decreased IL-10. TSA treatment partially reverses the effects even when PKM2 was silenced. SRSF3 silencing only decreased IL-10 under OGD, and did not affect other cytokines. TSA reduced IL-12 under normoxia and OGD, and TGF-β did so only under normoxic conditions. Therefore, SRSF3 and PKM2 pathways are at least partly involved in TSA's effects on cytokine production.

## Discussion

In the present study, we investigated the protective effects of TSA under hypoxic and glucose-deprivation conditions. TSA modulated the function and phenotype of DCs by upregulating critical costimulatory molecules in DCs associated with antigen-specific immune responses. CD80 and CD86 are two important surface markers for transducing T-cell activation signals. Hypoxia slightly reduced expression of CD86, and this was abolished by TSA treatment. CD80 and CD86 were upregulated under OGD, indicating a more mature phenotype. These data partially agree with a recent report evaluating another HDAC inhibitor LBG589 in DCs (Song et al., 2011), and in this study, CD80 was upregulated but CD86 was not affected.

Furthermore, hypoxia decreased endocytic activity, a specialized function of immature DCs (Sallusto et al., 1995). It has been reported that hypoxic conditions decrease the capability of immature DCs to uptake take up foreign antigens (Elia et al., 2008; Yang et al., 2009; Ogino et al., 2012). DCs were assessed for antigen uptake with GM-CSF/IL-4 stimulation under chronic hypoxic conditions, and there was marked inhibition of dextran, BSA, and LPS endocytosis compared to their counterparts under normoxia. This was accompanied by downregulation of C-type lectin receptors and reduced activation of the RhoA/Ezrin-Radixin-Moesin pathway (Elia et al., 2008; Yang et al., 2009). In line with these investigations, our observation coincides with the results from Jantsch's group (Jantsch et al., 2008), who showed that short-term hypoxia reduced the phagocytic activity of murine DCs generated under normoxia. These observations indicate that oxygen content is a critical for regulating DC function. Our data indicate that TSA may be a key to regulating DC maturation and T-cell activation.

Our model reflects an immune response under the deprivation of nutrients and oxygen. Immune cells recruited from high oxygen content blood flow to pathologic lesions with inflammatory conditions must rapidly adapt their metabolism to cope with reduced oxygenation (Sitkovsky and Lukashev, 2005). Hypoxia is generally anti-proliferative, and recent work suggests that acute hypoxia activates the cell death program in DCs (Sitkovsky and Lukashev, 2005; Carraro et al., 2007; Naldini et al., 2012). As oxygen becomes limited, transcription factor HIF-1α (Denko, 2008) stabilizes and drives the expression of genes involved in compensatory pathways, whose functions include restoring oxygen delivery and maintaining energy requirements, which promote cellular survival and proliferation (Cummins and Taylor, 2005; Muz et al., 2009; Prabhakar and Semenza, 2015). HIF-1α is also a key factor in the immune function of DCs. Under normoxia, LPS stimulates more HIF-1α expression in DCs (Jantsch et al., 2008), which is associated with elevated expression of HIF1-α target genes. In HIF-1α-deficient DCs, induction of the costimulatory molecules CD80 and CD86 was attenuated when stimulated by LPS, and these DCs are less capable of driving T-cell proliferation (Jantsch et al., 2008; Bhandari et al., 2013).

Dendritic cells undergo metabolic reprogramming, as they upregulate glycolysis/gluconeogenesis and pentose phosphorylation genes (Cummins and Taylor, 2005; Muz et al., 2009; Prabhakar and Semenza, 2015). HIF1-α induces the expression of many enzymes in the glycolysis pathway (Semenza et al., 1991, 2000; Palsson-McDermott and O'Neill, 2013), such as HK2, TPI-1, and LHDA (Luo et al., 2011). Glycolysis is vital for survival and ATP production in the absence of mitochondrial ATP generation. During aerobic glycolysis, pyruvate, the end product of glycolysis, does not feed into the TCA cycle to boost subsequent OXPHOS but is instead metabolized to lactate. Thus, a hallmark of anaerobic glycolysis is increased lactate production. In our study, TSA treatment restored ATP and lactate, which were decreased by oxygen or glucose deprivation, indicating beneficial effects. In contrast, TSA increased mRNA of HIF-1α as well as its downstream target genes HK2, LDHA and TPI-1. Thus, TSA may promote an HIF-1α-dependent glycolytic pathway and improve DC2.4 cell survival.

As immunomodulators, DCs secrete numerous cytokines or chemokines, which modulate the balance between adaptive immunity and inflammatory response (Banchereau and Steinman, 1998; Steinman, 2003; Joffre et al., 2009). When exposed to pathogens, or danger-associated molecular patterns (DAMPs) generated by hypoxia-induced cell death via Toll-like receptors (TLRs), DCs can produce pro-inflammatory cytokines, which form a niche favoring specific T cell expansion (Napolitani et al., 2005). Under hypoxia, human monocyte-derived DCs upregulated IL-10 (Song et al., 2011) and pro-inflammatory cytokine IL-1β (Mancino et al., 2008), which is functionally important for T-cell priming. IL-12 is involved in Th1 polarization (Lyakh et al., 2008), whereas TGF-β is important for T-cell development and DC tolerance (Esebanmen and Langridge, 2017).

We compared cytokine expression under normal conditions, hypoxia, and hypoxia and OGD. Cytokine production was altered by hypoxia, and glucose deprivation alone had no effect on cytokines. IL-12 and TGF-β were attenuated by GD, and cytokines were upregulated by OGD. Our findings differ from recent findings showing that hypoxia influences IL-1β and IL-10 expression (Mancino et al., 2008; Song et al., 2011), likely due to differences in HDACI type, concentrations, and exposure time. Reduced expression in cytokines is not due to decreased protein expression under hypoxia because all cytokines tested in our experiment were increased under OGD. Furthermore, HDAC TSA significantly reduced cytokine expression under GD and OGD in most cases. This result, however, is consistent with several previous reports. Nencioni's group tested the effect of HDACI MS-275 and TNFα; IL-6 and IL-12 secretion decreased, as did secretion of anti-inflammatory cytokine IL-10 under poly (I-C) (Nencioni et al., 2007). Moreover, in murine DCs, HDACIs TSA and VPA were shown to repress secretion of pro-inflammatory cytokines TNFα, IL-1β, IL-6, and IL-12 (Bode et al., 2007; Frikeche et al., 2012b). Thus, our findings indicate that TSA may modulate the immune system by regulating DC-derived cytokines, which modulate T-cell polarization and reduce inflammation.

Pyruvate kinase M2 (PKM2) has been shown to promote rapid glycolytic energy production. It is generally upregulated in cancer cells and is important for metabolic programming in immune cells under LPS stimulation, so it contributes to activation of macrophages and DCs (Altenberg and Greulich, 2004; Palsson-McDermott et al., 2015). PKM2 dimers can translocate to the nucleus, where they directly interact with HIF-1α to regulate the expression of the pro-glycolytic enzymes GLUT1, PHD3, PDK1, LDHA, and PKM2 (Mancino et al., 2008; Wang et al., 2012). The enzymatically active PKM2 tetramers remain in the cytoplasm, supporting the final rate-limiting conversion from phosphoenolpyruvate (PEP) to pyruvate (Mazurek et al., 2005). PKM2 expression is transcriptionally activated by HIF-1α under hypoxia (Mancino et al., 2008). Pyruvate kinase isoforms PKM1 and PKM2 are alternatively spliced products of the *PKM2* gene (Mancino et al., 2008). In cancer cells, serine/arginine-rich protein (SRSF3) activates exon 10 expression, which favors expression of PKM2 (Carraro et al., 2007).

Here, we show that TSA treatment favors the expression of PKM2 over PKM1 mRNA and protein, and SRSF3 expression was upregulated by TSA. Silencing PKM2 or SRSF3 via shRNA confirmed our hypothesis that SRSF3 acts upstream of PKM2. Furthermore, TSA could partially rescue the effects of PKM2 or SRSF3 silencing on ATP and lactate production. Finally, the interplay between PKM2 and SRSF3 also affected IL-1β, IL-10, IL-12, and TGF-β. These findings suggest that TSA increases PKM2 expression via upregulation of SRSF3, thus improving DC 2.4 cell glycolytic activity and function.

In summary, our findings suggest that TSA significantly modulated the phenotype and function of murine DCs under hypoxia and glucose deprivation, suggesting a therapeutic immunomodulatory role for HDAC inhibitors.

## Author contributions

HJ and SZ conducted the experiments. HJ and SZ contributed equally to the project. SZ and XC designed the experiments and SZ wrote the paper. TS, XG, and RZ helped with some of the experiments.

### Conflict of interest statement

The authors declare that the research was conducted in the absence of any commercial or financial relationships that could be construed as a potential conflict of interest.
